# A theranostic abscisic acid-based molecular glue†

**DOI:** 10.1039/d2sc06995d

**Published:** 2023-02-28

**Authors:** Jing Chen, Huong T. X. Nguyen, Ming Yang, Fangxun Zeng, Hang Xu, Fu-Sen Liang, Wei Wang

**Affiliations:** a Department of Pharmacology and Toxicology, R. Ken Coit College of Pharmacy, University of Arizona 1703 E. Mabel Street P. O. Box 210207 Tucson AZ 85721-0207 USA weiwang1@arizona.edu; b Department of Chemistry, Case Western Reserve University 2080 Adelbert Road Cleveland Ohio 44106 USA fxl240@case.edu

## Abstract

Molecular glues, capable of selectively controlling the interactions between specific pairs or groups of proteins and the associated downstream effects, have become a promising strategy for manipulating cellular functions and developing novel therapies for human diseases. Theranostics with both diagnostic and therapeutic capabilities acting at disease sites has become a powerful tool to achieve both functions simultaneously with high precision. To selectively activate molecular glues at the desired site and monitor the activation signals at the same time, here we report an unprecedented theranostic modular molecular glue platform integrating signal sensing/reporting and chemically induced proximity (CIP) strategies. We have demonstrated for the first time the integration of imaging and activation capacity with a molecular glue on the same platform to create a theranostic molecular glue. A theranostic molecular glue ABA-Fe(ii)-F1 was rationally designed by conjugating a NIR fluorophore dicyanomethylene-4*H*-pyran (DCM) with a CIP inducer abscisic acid (ABA) using a unique carbamoyl oxime linker. We have also engineered a new version of ABA-CIP with an enhanced ligand-responding sensitivity. We have validated that the theranostic molecular glue can sense Fe^2+^ and produce turn-on NIR fluorescence for monitoring as well as releasing the active inducer ligand to control cellular functions including gene expression and protein translocation. This novel molecular glue strategy paves the way to building a new class of molecular glues with theranostic capacity for research and biomedical applications.

## Abstract 2

A theranostic ABA-based molecular glue, capable of sensing Fe(ii) and triggering the release of ABA and NIR fluorophores, has been developed for spatiotemporal monitoring and manipulating cellular functions.

## Graphical abstract

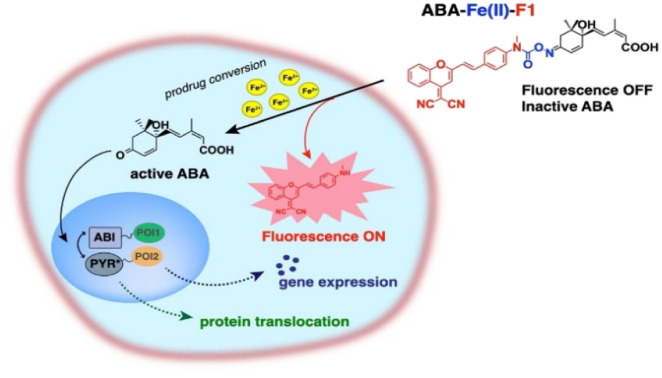

## Introduction

Molecular glues control the proximity between two (or more) proteins of interest (POIs) and the artificially triggered associations between the chosen POIs lead to dedicated biological effects and offer an unparalleled power to alter the protein interactome and cellular circuitry for new cellular functions.^1^ Certain molecular glues, either naturally occurring (*e.g.*, FK506) or synthetic (*e.g.*, proteolysis targeting chimeric (PROTAC)), can induce proximity between endogenous proteins (*e.g.*, FKBP12-calcineurin or E3 ubiquitin ligase-POI) to trigger pre-defined effects (*e.g.*, suppressing immune response or promoting protein degradation). However, molecular glues targeting endogenous proteins are either discovered serendipitously or can be difficult to develop. To expand the utility of molecular glues for broader applications controlling proximity between a wide range of cellular proteins, chemically induced proximity (CIP) technology has been developed that uses molecular glues to promote the dimerization of two unique inducer-binding adaptor proteins that are fused individually to two chosen POIs leading to desired biological and therapeutic functions.^2,3^ For example, plant-originated abscisic acid (ABA) heterodimerizes PYL/PYR- and ABI-fusion proteins,^4^ while gibberellic acid (GA) can trigger the dimerization of GID1 and GA.^5^ Depending on the choice of POIs in the engineered CIP fusion protein components, a variety of downstream biological events can be specifically manipulated by these molecular glues, which provide a rapid and modular way to create novel molecular glue/CIP-responsive synthetic cellular functions.^6–8^ This novel molecular glue-based induced protein complexation strategy has emerged as a promising technology for manipulating biological processes and developing therapeutic agents.^9^

Achieving high specificity in activating therapeutic effects at desired locations and time is critical for successful disease treatment and prevent side effects, which however still presents daunting challenges. Although current molecular glues and CIP methods can achieve excellent temporal control,^6^ the spatial control (*i.e.*, the locations where the triggered effects take place) is lacking, which typically is addressed by using special delivery methods (*e.g.*, nanoparticles and viral vectors) to selectively deliver the inducers or the CIP gene or protein components to the desired sites.^10^ Theranostic prodrugs equipped with fluorophores as reporters enable the monitoring of the drug delivery and the sites of action, which significantly contribute to precision therapy.^11–14^ Herein, we report the application of the theranostic prodrug strategy combined with the CIP platform to generate novel molecular glue entities. This new class of theranostic molecular glues not only monitor the presence of specific biological or pathological stimuli, but also activate CIP-controlled effects at the site of signal stimulation to achieve the spatiotemporal control of the cellular or therapeutic functions and processes. A novel theranostic molecular glue, ABA-Fe(ii)-F1, was rationally constructed by conjugating a NIR fluorophore dicyanomethylene-4*H*-pyran (DCM) with ABA using a unique carbamoyl oxime linker as a caging and sensing unit for Fe^2+^, which has played critical roles in biology and disease development. We demonstrated that ABA-Fe(ii)-F1 in biological settings displayed an off–on fluorescence signal in the presence of Fe^2+^ and concurrently triggered the release of ABA. The activated ABA can subsequently promote the dimerization of a new pair of engineered, more sensitive PYR- and ABI-fusion proteins to induce cellular processes and functions. This new synthetic biotechnology and modular molecular glue platform can be modified for responding to other endogenous biological signals to control different effects and holds great potential for various theranostic applications.

## Results and discussion

### Design and synthesis of Fe^2+^ responsive theranostic molecular glue ABA-Fe(ii)-F1

We have demonstrated in our prior work the feasibility of caging the ABA carboxylate as an ester in a prodrug form to activate and release active ABA by light or signalling molecules H_2_O_2_ and Fe^2+^.^15–17^ However, the spatial location of the responding signal as well as ABA prodrug activation cannot be monitored in previous methods. We aimed to develop new theranostic ABA-derived molecular glues that can respond to specific signalling molecules and simultaneously trigger the release of uncaged ABA, which should produce fluorescence for the imaging of the chosen cellular or disease signal and the monitoring of ABA activation. In the study, we are particularly interested in Fe^2+^-responsive ABA molecular glues. Iron [Fe^2+^/Fe^3+^] is the most abundant and essential transition metal in the human body and plays important biological roles.^18–21^ Its redox balance is critical for oxygen^22^ and DNA/RNA metabolisms,^23,24^ heme synthesis,^25^ and neural activities.^26^ The emerging ferroptosis, an iron-dependent phospholipid peroxidation-induced cell death process, reveals the new roles of iron species in physiology and pathology.^27^ Although most iron ions exist in complexes with proteins, free Fe^2+^ is present in cellular environments where it is believed to participate in critical physiological functions.^28^ An elevated level of Fe^2+^ has been linked with Alzheimer's and Parkinson's diseases.^29^ Therefore, developing a theranostic molecular glue system that enables monitoring of elevated levels of Fe^2+^ in local environments and in the meanwhile producing specific subsequent biological effects for cellular function studies or therapeutic interventions is particularly desirable. Several Fe^2+^ fluorescent imaging probes have been developed but are largely limited to the detection or monitoring of the levels of Fe^2+^ in biological systems without the capability to simultaneously perturb biological functions.^30–36^

**Scheme 1:**
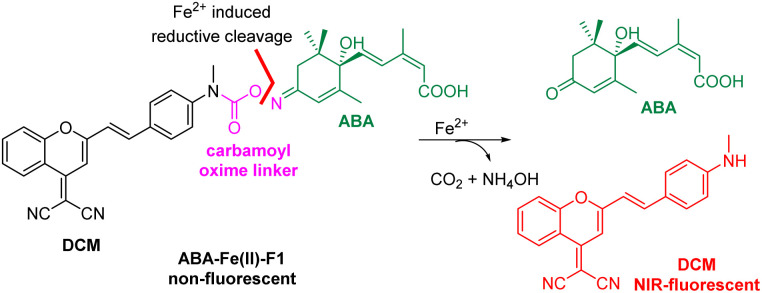
Design of Fe^2+^ responsive theranostic ABA molecular glue ABA-Fe(ii)-F1.

In the design of Fe^2+^-responsive theranostic ABA-based molecular glues, several factors should be taken into consideration. Our previous studies relied on caging the ABA carboxylate as an ester,^15,16^ since it is critical for binding to the PYL/PYR adaptor protein and is easy to chemically modify.^37^ However, the ester groups have been known to be sensitive to esterase-mediated hydrolysis in cells and *in vivo*, thus can generate undesired background reactions. To overcome the issue, we seek to identify a new site to mask ABA. The examination of ABA interaction with PYL1 reveals that the ketone also provides a critical contact with Arg143 *via* a H_2_O-engaged hydrogen bond network.^37^ We therefore believe that the ketone on ABA can serve as a new caging site to abolish ABA-mediated ABI-PYL/PYR dimerization activity. An oxime could be a viable caging group (Scheme 1), since it has been used to cage ketones in bioorthogonal reactions and show excellent biocompatibility and stability in the biological environment.^38^ In order to engineer the oxime chemistry into theranostic ABA molecular glues, we explored new oxime containing linker structures that connect ABA with an off–on fluorophore. The linker should effectively cage the ketone group on ABA, while also serving as a sensing unit for selectively responding to Fe^2+^-mediated cleavage to turn on the fluorophore for imaging and release ABA for functional perturbation. We expect that a new carbamoyl oxime linker could meet the demand. The electron withdrawing capacity of the carbamoyl moiety can make the oxime N–O bond become active enough for the Fe^2+^-mediated reductive cleavage^39^ and at the same time mask the electron-donating amine group in the fluorophore to offer the desired off–on fluorescence signal. The DCM dye was selected as the fluorophore in our theranostic molecular glue design. It exhibits emission in the near infrared (NIR) range (*λ*_em_ 685–720 nm), with a large Stokes shift (>180 nm) and high photostability,^40,41^ thus making it appealing for biological imaging. It is noted that NIR fluorophores as the imaging modality can penetrate deeper into the tissue with minimal phototoxicity/tissue damage and low background interference.^42^ Caging the amino group in DCM by the carbamoyl group could shift its property from electron-donating to electron-withdrawing, thus blocking internal charge transfer (ICT) to give a low background fluorescence signal. It is expected that Fe^2+^-induced reduction of carbamoyl oxime triggers the fragmentation to release ABA and byproducts NH_4_OH and DCM with concurrent formation of CO_2_ with off–on fluorescence. The released free ABA can trigger PYL/PYR and ABI binding to induce desired biological functions.

**Scheme 2:**
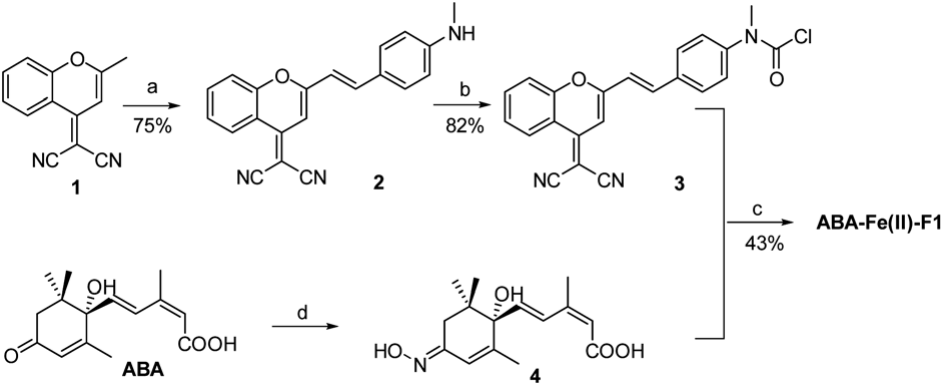
Synthesis of the theranostic probe ABA-Fe(ii)-F1. Reagents and conditions: (a) 4-(methylamino)benzaldehyde, AcOH, piperidine, toluene, reflux, and 5 h; (b) triphosgene, DCM, rt, and 12 h; (c) 4, pyridine; (d) hydroxylamine, 4 Å molecular sieves (MSs), pyridine, reflux, N_2_, and 12 h.

The synthesis of the theranostic molecular glue, ABA-Fe(ii)-F1, is briefly described in Scheme 2. The 2-(2-methyl-4*H*-chromen-4-ylidene)malononitrile 1 was synthesized from a commercially available starting material according to a reported procedure.^40^ The resulting 1 was converted to the NIR fluorophore DCM 2 through a condensation with 4-(methylamino)benzaldehyde. DCM 2 was then treated with triphosgene to afford carbamic chloride 3. ABA was reacted with hydroxylamine to give oximine 4 quantitatively, followed by coupling with carbamic chloride 3 to yield the ABA-Fe(ii)-F1 theranostic probe.

### Study of the reactivity and selectivity of ABA-Fe(ii)-F1 toward Fe^2+^

**Fig. 1:**
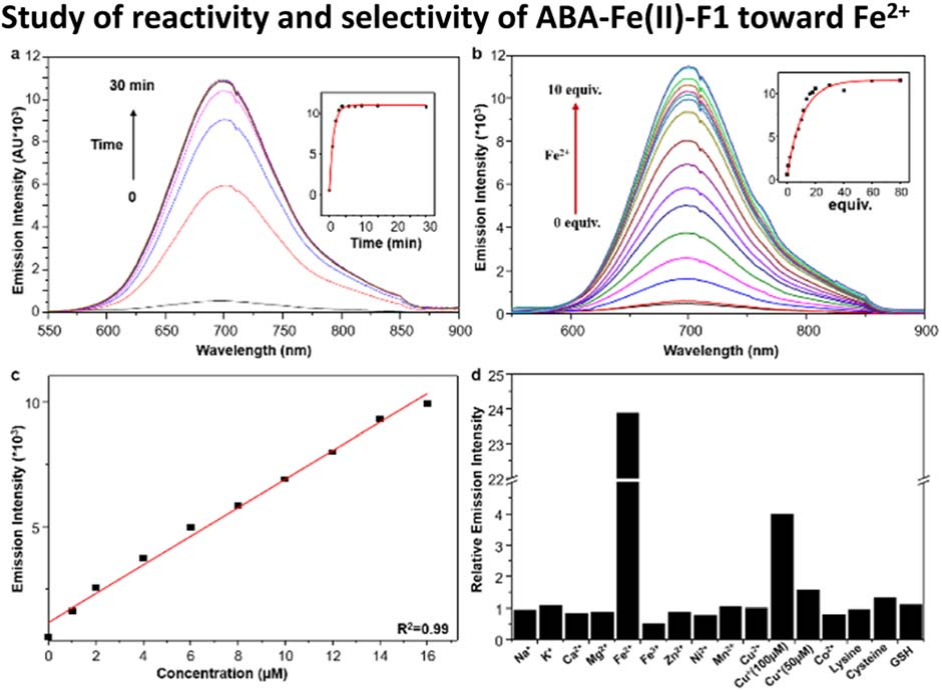
(a) Fluorescence emission of the 10 μM ABA-Fe(ii)-F1 probe in 50% HEPES/DMSO (10 mM HEPES, pH 7.4) after reaction with 100 μM Fe^2+^ at times of 0, 1, 2, 3, 4, 6, 8, 10, 15, and 30 min. Change of emission intensity at 700 nm is shown in the inset. (b) Fluorescence response of 10 μM ABA-Fe(ii)-F1 to increased concentration of Fe^2+^ (0–10 equiv.) in 50% HEPES/DMSO (10 mM HEPES, pH = 7.4). (c) Low detection limit of the 10 μM ABA-Fe(ii)-F1 probe to Fe^2+^. (d) Fluorescence response of 10 μM ABA-Fe(ii)-F1 to biologically relevant d-block (100 μM) and s-block (1 mM) metals as well as lysine, cysteine and glutathione (GSH) (5 mM).

With ABA-Fe(ii)-F1 in hand, its fluorescence response to Fe^2+^ was evaluated in 50% HEPES/DMSO aqueous buffer (10 mM HEPES, pH 7.4). ABA-Fe(ii)-F1 showed absorption maxima at 438 nm, and emission maxima at 700 nm, with a Strokes shift of 262 nm. Upon the exposure to Fe^2+^, ABA-Fe(ii)-F1 exhibited an increase in fluorescence emission at 700 nm (Fig. 1a). Notably, this theranostic probe responded to Fe^2+^ very rapidly. The increase in fluorescence intensity reached a plateau within 5 min when 10 μM ABA-Fe(ii)-F1 was treated with 100 μM Fe^2+^ in HEPES buffer. The intensity increase displayed a Fe^2+^ concentration dependent manner and a high sensitivity (Fig. 1b). As low as a 0.9 μM level of Fe^2+^ could be detected, which is more sensitive than another Fe^2+^ sensing probe that we have reported earlier.^26^ The high sensitivity of the probe offers a capacity for sensing Fe^2+^ in biological systems.^43^ Furthermore, a linear correlation between fluorescence intensity and Fe^2+^ concentration was observed (Fig. 1c). This feature allows for the qualification of Fe^2+^.

We next evaluated the selectivity of ABA-Fe(ii)-F1 for Fe^2+^ by testing a panel of biologically relevant transition, alkali, and alkaline earth metals (Fig. 1d). We observed that ABA-Fe(ii)-F1 exhibited a highly metal- and oxidation state-specific response to Fe^2+^. It gave negligible fluorescence turn-on in the presence of cysteine and glutathione, the abundant major intracellular reductants. Only Cu^+^ at 100 μM levels induced a modest response, but ABA-Fe(ii)-F1 was not responsive to lower concentrations of Cu^+^ (50 μM or less). These data, combined with the 10-fold higher abundance of iron over copper in typical eukaryotic cells,^44,45^ and the relatively high buffering capacity for cellular copper in the form of glutathione and metallochaperones,^46–49^ suggested that ABA-Fe(ii)-F1 had a sufficient *in vitro* selectivity profile for applications in labile iron detection in biological systems.

### Investigation of Fe^2+^ triggered ABA release from ABA-Fe(ii)-F1

**Fig. 2:**
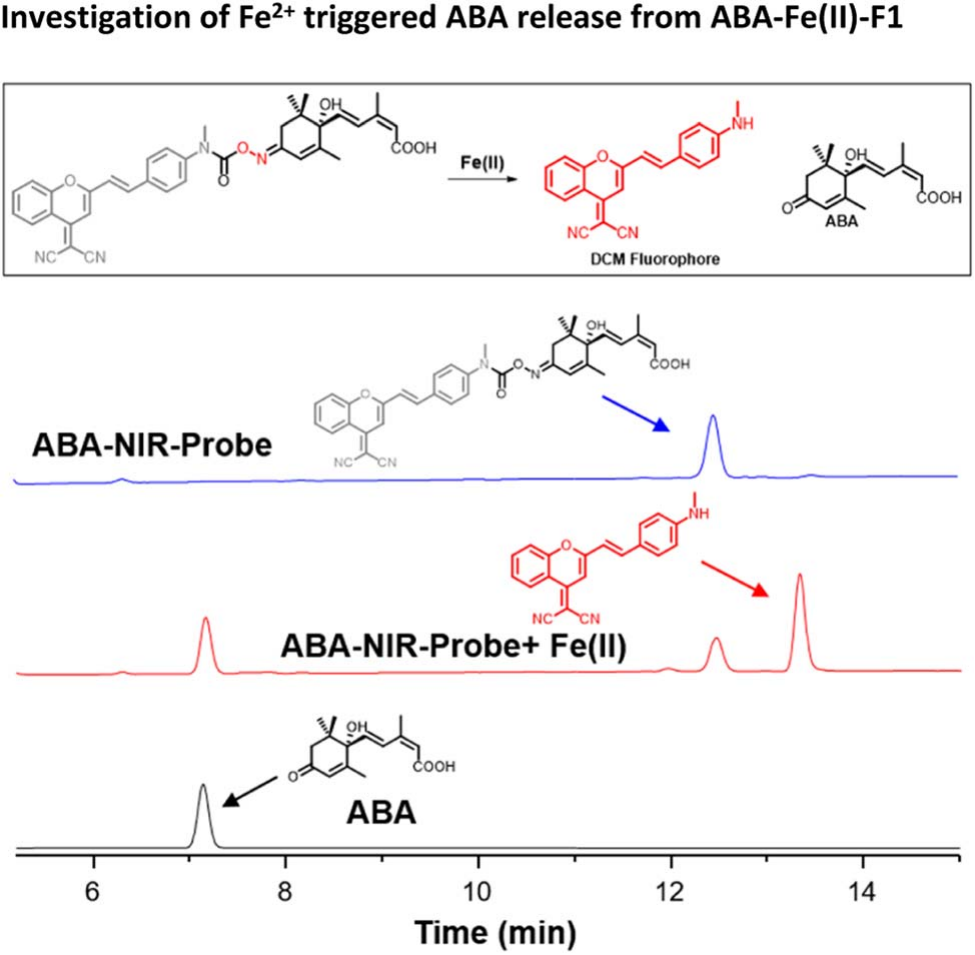
The UPLC analysis of reactions of ABA-Fe(ii)-F1 (10 μM) with Fe^2+^ (100 μM) at 37 °C in 50% DMSO/HEPES buffer for 30 min.

Next, we examined the Fe^2+^-triggered release capacity of ABA by incubating 10 μM ABA-Fe(ii)-F1 with 100 μM Fe^2+^ in 50% HEPES/DMSO aqueous buffer (10 mM HEPES, pH 7.4) at 37 °C. The release event was monitored by ultra-performance liquid chromatography (UPLC). We observed that, without Fe^2+^, ABA-Fe(ii)-F1 stayed intact and no ABA was produced during the observation period (Fig. 2). Upon the addition of Fe^2+^, ABA-Fe(ii)-F1 was converted to ABA within 30 min. Following the cleavage process using UPLC, we also observed that the DCM fluorophore peak appeared, confirmed by the prepared DCM as a reference. The studies validated our working hypothesis that ABA-Fe(ii)-F1 was sensitive to Fe^2+^ and led to the release of ABA and the concomitant generation of the DCM fluorophore.

**Fig. 3:**
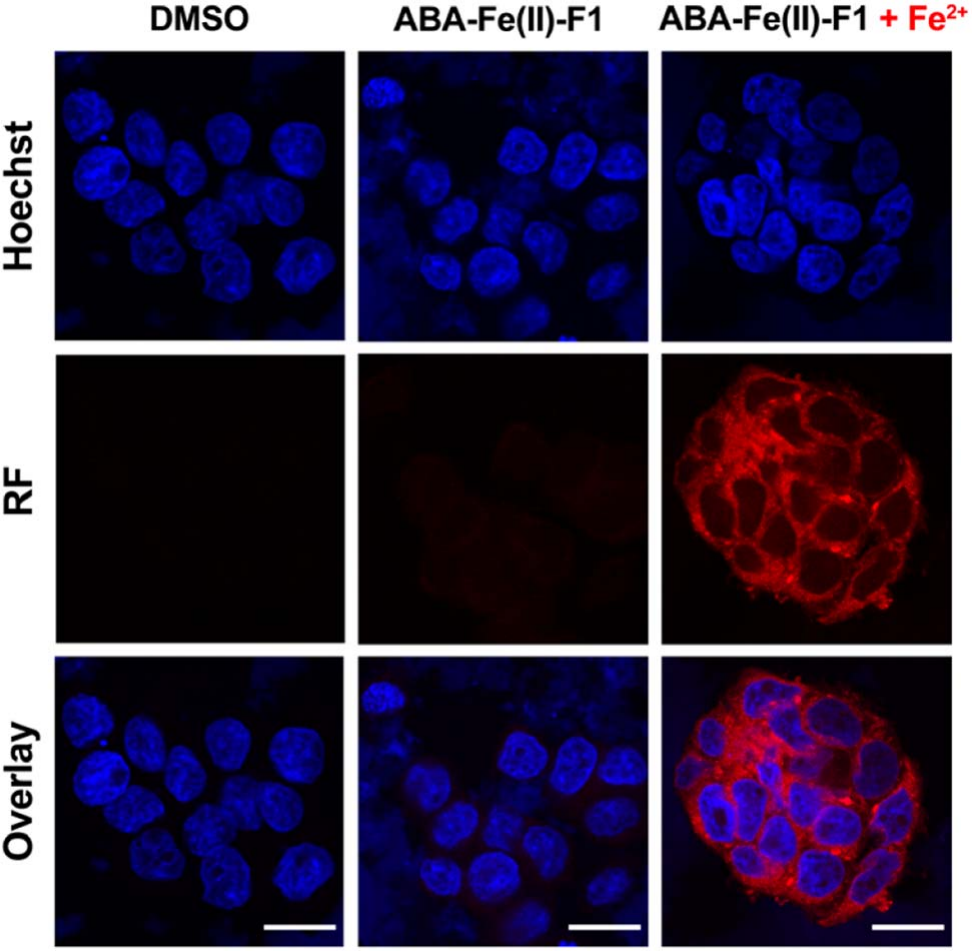
Fe^2+^-triggered “turn-on” red fluorescence from ABA-Fe(ii)-F1 in cells. HEK-293T cells treated with DMSO or ABA-Fe(ii)-F1 with or without Fe^2+^ for 30 min. Cells were stained with Hoechst 33 342 for nuclei and red fluorescence reported the presence of Fe^2+^. Imaging channels: Hoechst: *λ*_ex_ = 405 nm and *λ*_em_ = 415–485 nm. Red fluorescence (RF): *λ*_ex_ = 514 nm and *λ*_em_ = 685–720 nm. Scale bar = 20 μM. Shown are representative confocal fluorescence images from 3 independent experiments.

Next, we evaluated the effectiveness of ABA-Fe(ii)-F1 to sense Fe^2+^ in the living cell system and to report it through generating a red fluorescence signal. HEK-293T cells were incubated with 10 μM ABA-Fe(ii)-F1 (or DMSO as a negative control) for 1 h to ensure the compound loading in cells. The culture media were then replaced with fresh ones without ABA-Fe(ii)-F1 to remove any remaining extracellular compound, followed by treating cells with 5 mM Fe^2+^ for 30 min. The cells were then fixed and analyzed under a confocal fluorescence microscope. We observed a significant increase in the intracellular far-red fluorescence signal only in ABA-Fe(ii)-F1 and Fe^2+^-treated cells, whereas no discernible fluorescence signal was detected in cells without Fe^2+^ or ABA-Fe(ii)-F1 (Fig. 3).

**Fig. 4:**
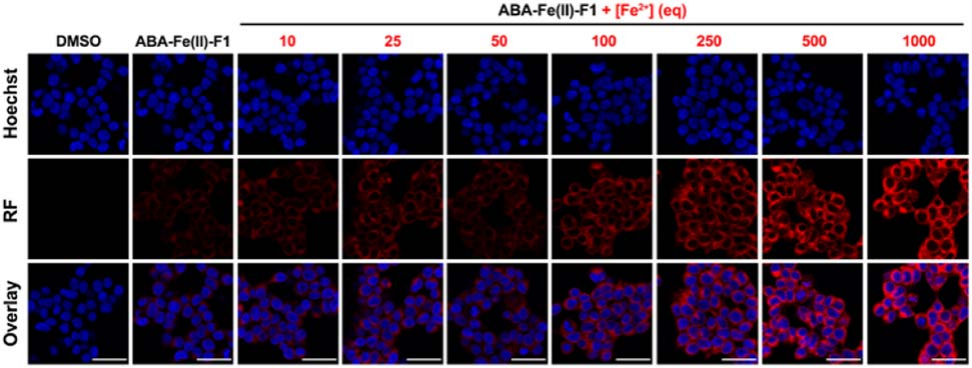
The dosage response of ABA-Fe(ii)-F1 to Fe^2+^ in cells. HEK-293T cells incubated with ABA-Fe(ii)-F1 and varying concentrations of Fe^2+^ for 30 min. The red fluorescence generated by ABA-Fe(ii)-F1 from Fe^2+^ stimulation was observed under a confocal microscope. Cell nuclei were stained with Hoechst (blue). Images shown are representative images from 3 independent biological experiments. Scale bars = 40 μm.

**Fig. 5:**
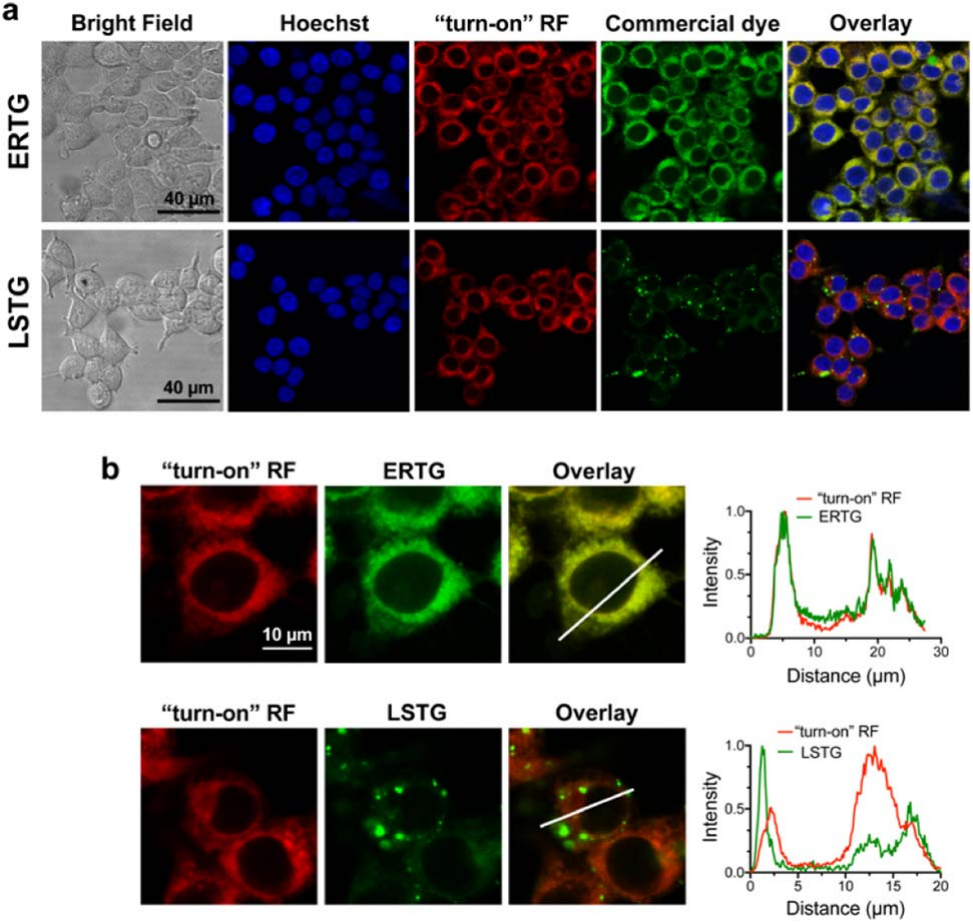
Sub-cellular localization of ABA-Fe(ii)-F1 generated red fluorescence signals in cells. (a) HEK-293T cells were treated with ABA-Fe(ii)-F1 (10 μM) and Fe^2+^ (5 mM) and co-stained with Hoechst (blue, for nuclei) or commercial dyes staining sub-cellular organelles including ERTG (6 μM) for the ER and LSTG (500 nM) for the lysosome. The “turn-on” RF panel shows the released DCM fluorescence. The representative images show the staining with individual dyes and the overlay images show merging of different staining. Scale bars = 40 μm. (b) Analyses of the overlay of probe-generated fluorescence with lysosome and ER staining dyes. The intensity profiles of the region of interest (ROI) across the cell from each dye and red fluorescence signals were obtained by using ImageJ. Imaging parameters: Hoechst: *λ*_ex_ = 405 nm and *λ*_em_ = 415–485 nm; RF: *λ*_ex_ = 514 nm and *λ*_em_ = 685-720 nm; ERTG and LSTG: *λ*_ex_ = 496 nm and *λ*_em_ = 510–560 nm. Scale bar = 10 μm. Images shown are representative images from 3 independent biological experiments.

After confirming that ABA-Fe(ii)-F1 could successfully sense and report the presence of Fe^2+^, we tested different concentrations of ABA-Fe(ii)-F1 in response to Fe^2+^ in cells and found that 5 μM of the compound was sufficient to give significant fluorescence outputs while 10 μM offered even higher signals (Fig. S1†). To investigate the dosage response of ABA-Fe(ii)-F1 to different concentrations of Fe^2+^ (from 10 to 1000 equiv.) in cells, HEK-293T cells were pre-treated with 10 μM ABA-Fe(ii)-F1 for 1 h as described above, followed by the addition of Fe^2+^ at indicated concentrations. After 30 min, the fluorescence signals in cells were monitored under a confocal fluorescence microscope (Fig. 4) and the red fluorescence intensity was also quantified (Fig. S2 and S3†). We found that the signals of fluorescence intensity were Fe^2+^ concentration dependent. The intensity reached a maximum level around 500 to 1000 equiv. of Fe^2+^. Interestingly, we observed a distinct cellular distribution of the fluorescence signal, which prompted us to examine its subcellular localization. A series of co-staining experiments were performed in different cell lines, including HEK-293T, HeLa and MDA-MB-231, by treating cells with 10 μM ABA-Fe(ii)-F1 and 5 mM Fe^2+^ for 30 min and staining cells with commercial organelle-specific fluorescence dyes, including lysosome specific LysoTracker Green (LSTG) and endoplasmic reticulum (ER) specific ER-tracker Green (ERTG) dyes. The staining results were observed under a confocal fluorescence microscope and subsequent analyses revealed that the fluorescence generated by ABA-Fe(ii)-F1 was selectively localized to the ER (Fig. 5, and S4†). Furthermore, to verify that the observed ER localization was an intrinsic property of the released fluorescence moiety (*i.e.*, DCM) from ABA-Fe(ii)-F1, instead of any influence from the ABA structure, the co-localization of the respective commercial ER and lysosome dyes with the fluorophore DCM was also examined. As shown in Fig. S5,† the fluorescence of DCM perfectly overlapped with the ERTG staining, confirming the ER-localization of the released fluorophore.

### Engineering the ABA CIP protein component with enhanced sensitivity for the theranostic molecular glue ABA-Fe(ii)-F1 and programming the inducible effects by ABA-Fe(ii)-F1 and Fe^2+^

**Fig. 6:**
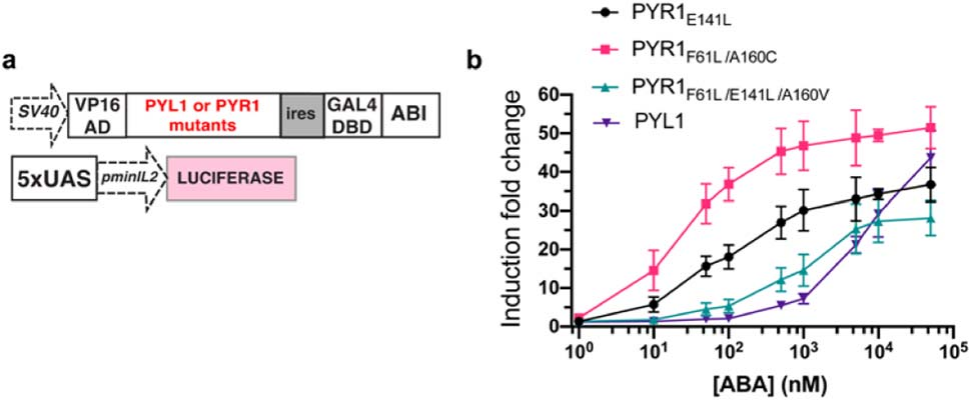
Engineering hypersensitive ABA-based CIP protein components. (a) DNA constructs for an ABA inducible luciferase expression containing PYL1 or different PYR mutants. (b) ABA-dependent luciferase expression using a split transcriptional activator pairing ABI with PYL1 or different PYR mutants. Induction fold changes were calculated by normalizing to the background level of DMSO-treated cells. Error bars represent mean ± SEM (*n* = 4).

To maximize the downstream effects induced by ABA-Fe(ii)-F1 after sensing Fe^2+^, we first engineered a new ABA CIP system with enhanced sensitivity. Due to the low affinity of ABA binding to PYL1, the current ABA-based CIP system in mammalian cells typically requires around 100 μM to induce consistent maximum functional effects. The 10 μM optimal concentration of ABA-Fe(ii)-F1 that we identified for Fe^2+^ imaging and reporting purposes may not produce satisfactory biological outputs through CIP. A recent study showed that mutations in PYR1 can affect ABA binding and the physiological outcomes in plants.^50^ To engineer a more potent pair of ABA CIP protein components accompanying the theranostic ABA-Fe(ii)-F1 based platform, we applied an inducible gene expression system that uses ABA to induce the proximity of the ABI-fused yeast GAL4 DNA binding domain (GAL4DBD) to the PYL/PYR-fused herpes simplex virus VP16 transactivation domain (VP16AD). The ABA-induced reconstitution of functional transcriptional activator turns on the expression of the GAL4DBD-targeted gene, which was luciferase in the following study.^4^ We cloned different mutants of PYR1, including PYR1_E141L_, PYR1_F61L/A160C,_ and PYR1_F61L/E141L/A160V_ into the ABA-responsive split transcriptional activator that included the PYL/PYR-fused VP16AD and a separate ABI-fused GAL4 DNA binding domain (GAL4DBD (Fig. 6a).^50^ To test the effects of these mutants on an ABA-induced luciferase expression, we transfected HEK-293T cells with plasmids expressing different versions of the split transcriptional activators and the inducible luciferase reporter^4^ for 24 h and then treated the cells with different concentrations of ABA for another 24 h before the cells were harvested for luciferase assays. We identified that PYR1_F61L/A160C_ (termed PYR*) can induce a significantly higher induction than the construct with PYL1 at concentrations of 10 nM and higher while both eventually reached similar induction levels around 50 μM (Fig. 6b). We therefore adopted PYR* into the ABA-CIP system for the following functional studies of the theranostic ABA-Fe(ii)-F1 compound.

**Fig. 7:**
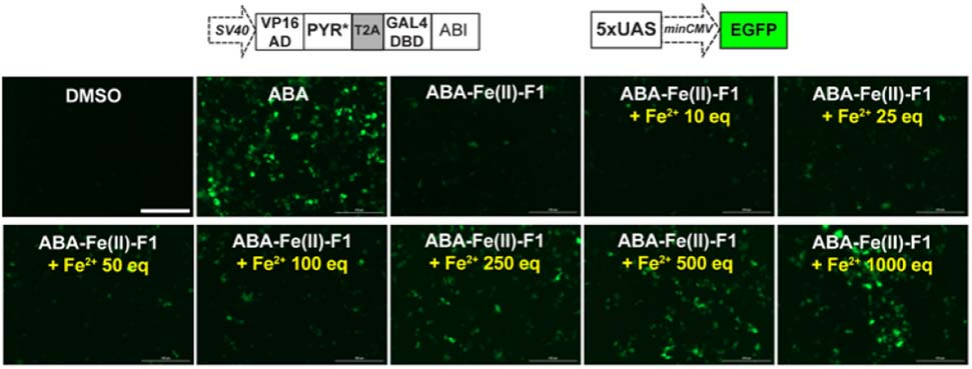
Fe^2+^-induced expression of EGFP mediated by ABA-Fe(ii)-F1 in cells. HEK-293T cells were transfected with plasmids encoding the ABA-inducible EGFP expression cassette for 24 h, and the cells were treated with 10 μM ABA, 10 μM ABA-Fe(ii)-F1 or ABA-Fe(ii)-F1 with varying concentration of Fe^2+^ for another 24 h. The EGFP expression induced by ABA-Fe(ii)-F1 in response to different Fe^2+^ concentrations as observed under a fluorescence microscope. Images shown are representative images from 3 independent biological experiments. Scale bars = 200 μm.

After we optimized the ABA-binding CIP protein components, we investigated the efficiency of ABA-Fe(ii)-F1 to generate biologically active ABA and induced a chosen downstream effect (*i.e.*, gene activation) in response to Fe^2+^. Using the ABA-inducible gene expression system described above, we cloned an ABA-inducible enhanced green fluorescence protein (EGFP) reporter gene cassette consisting of the split transcriptional activators containing VP16AD-PYR* and GAL4DBD-ABI linked by the T2A self-cleaving peptide, and an ABA-inducible EGFP gene under the control of a minimal promoter and 5 × UAS response element (Fig. 7). HEK-293T cells were transfected with these plasmids for 24 h and then treated with either 10 μM ABA (as a positive control) or 10 μM ABA-Fe(ii)-F1 with or without varying concentrations of Fe^2+^ for 24 h. The EGFP expression was observed under a fluorescence microscope and quantified using flow cytometry. Only in the presence of ABA, or ABA-Fe(ii)-F1 plus Fe^2+^ (but not without ABA or without Fe^2+^), we observed significant expression of EGFP (Fig. 7 and S6†), indicating the efficient generation of biologically active ABA from ABA-Fe(ii)-F1 by Fe^2+^, whereas the caged ABA-Fe(ii)-F1 compound itself cannot induce EGFP expression without Fe^2+^ stimulation. In addition, we also observed a dosage response of ABA-Fe(ii)-F1 towards Fe^2+^ levels with significant activations achieved at concentrations above 100 equiv. of Fe^2+^. The ABA-Fe(ii)-F1 compound was in general found to be relatively stable with a minimal EGFP induction even after 24 h incubation. We also tested the ABA-Fe(ii)-F1-mediated Fe^2+^-induced EGFP expression in other cell lines, including HeLa and CHO, and confirmed the effective induction of EGFP expression in these cell lines using the hypersensitive ABA CIP components (PYR*-ABI) (Fig. S7†).

### ABA-Fe(ii)-F1 simultaneously reported the presence of Fe^2+^ and induced protein translocation in cells upon Fe^2+^ stimulation

**Fig. 8:**
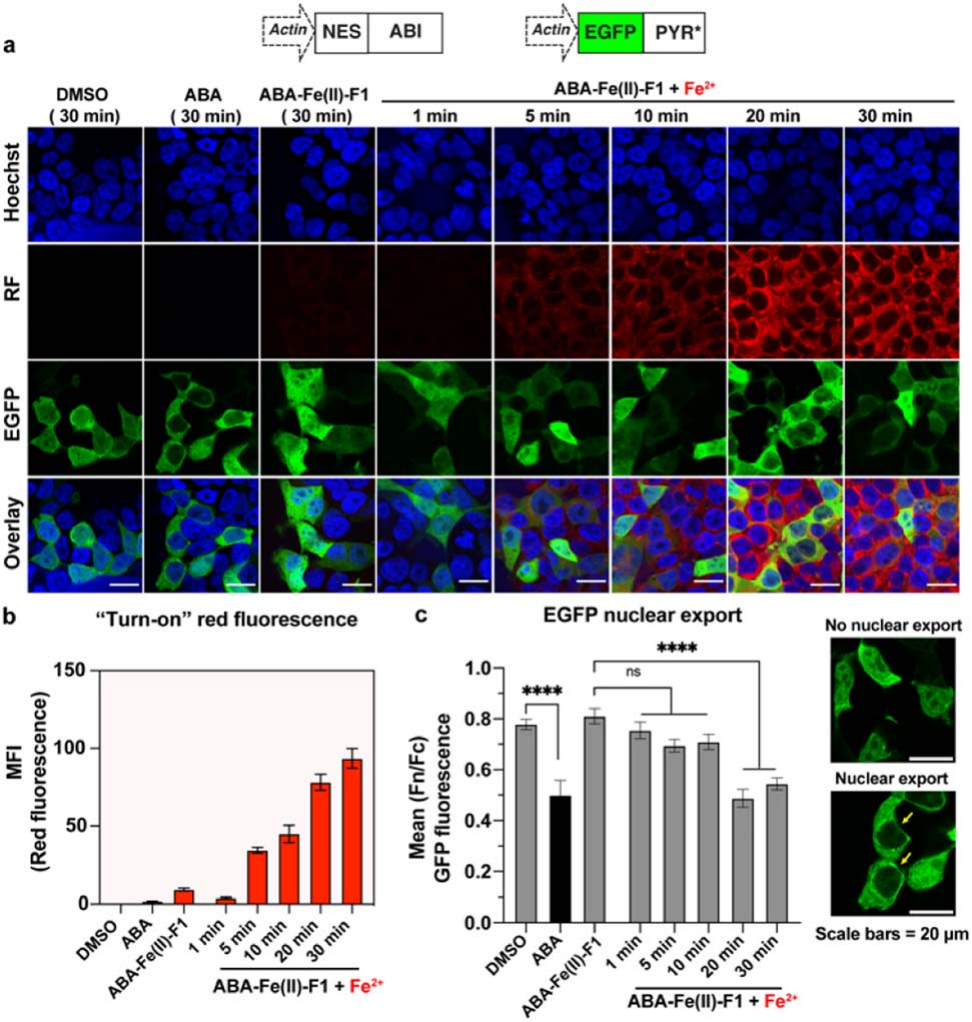
Fe^2+^-induced turn-on red fluorescence and nuclear export of EGFP in cells. (a) HEK-293T cells were transfected with inducible EGFP nuclear export plasmids for 16 h and the cells were treated with DMSO, ABA or ABA-Fe(ii)-F1 (10 μM) with or without Fe^2+^ (5 mM) for indicated time periods. Cell nuclei were stained with Hoechst (blue) and generated red fluorescence signals from released DCM are shown in the RF panel. The images were obtained using a confocal microscope and the representative images from 3 independent biological experiments. (b) The quantification of the mean fluorescence intensity (MFI) of red fluorescence in cells under the same experimental conditions as shown in (a). Data shown represent the mean ± S.E.M (*n* = 5). (c) (Left) The quantification of the mean nuclear export of EGFP in cells under conditions shown in (a). Images were analyzed to determine the ratio of nuclear-to-cytoplasmic (*F*_n_/*F*_c_) green fluorescence intensities. Error bars represent mean (*F*_n_/*F*_c_) ± SEM (*n* ≥ 20 individual cells, 2 independent experiments). ****: *p* ≤ 0.0001 and ns: *p* ≥ 0.05. Unpaired two-tailed Student's *t*-test was used to compared DMSO *vs.* the ABA group, one-way ANOVA with the Dunnett multiple comparison test was used to compared [ABA-Fe(ii)-F1 only] *vs.* Fe^2+^-treated groups, and (right) representative images of cells showing the nuclear export. Scale bars = 20 μm.

After we individually confirmed that ABA-Fe(ii)-F1 can respond to Fe^2+^ to generate an active fluorescent dye DCM for imaging and the functional ABA for inducing effects, we examined the effectiveness of using the ABA-Fe(ii)-F1 theranostic molecular glue for both functions simultaneously. We have previously showed that the ABA-induced nuclear export of EGFP occurred within minutes,^12^ which aligns with the timeframe of Fe^2+^-induced “turn-on” fluorescent probe release and will allow us to monitor both events – the reporting of the signal and the induction of the downstream cellular effect within the same time period. To achieve this, we cloned DNA constructs expressing ABI fused to a nuclear export sequence (ABI-NES) and EGFP fused to PYR* (EGFP-PYR*). HEK-293T cells were transfected with these plasmids for 16 h and then incubated with 10 μM ABA or 10 μM ABA-Fe(ii)-F1 with or without 5 mM Fe^2+^, for different time periods (1 to 30 min). At the indicated time points, cells were fixed and imaged under a confocal fluorescence microscope. We started to observe a significant increase of fluorescence from the DCM dye after 5 min of Fe^2+^ treatment when ABA-Fe(ii)-F1 was added (but not with ABA or ABA-Fe(ii)-F1 without Fe^2+^), which continued to increase during the 30 min monitoring period (the RF panel in Fig. 8a and b). These results again confirmed the imaging and reporting function of the theranostic ABA-Fe(ii)-F1 molecule. For its function in inducing biological effects, we analyzed the Fe^2+^-dependent EGFP fusion protein translocation in cells. The EGFP-PYR* fusion protein was distributed within the entire cell before induction. After the ABA-Fe(ii)-F1-treated cells were incubated with Fe^2+^ for 20 min, we observed a clear nuclear export of EGFP at a level similar to the translocation induced by free ABA under a fluorescence microscope (the EGFP panel in Fig. 8a) as well as from the quantification of the nuclear-to-cytoplasmic shift of fluorescence signal yields (Fig. 8c). These data indicated that caging the ketone moiety in ABA was an effective approach to abolish ABA dimerization activity and generate an effective Fe^2+^-responsive and biologically stable theranostic ABA-Fe(ii)-F1 molecular glue for simultaneously producing turn-on fluorescence and inducing CIP-mediated effects.

**Fig. 9:**
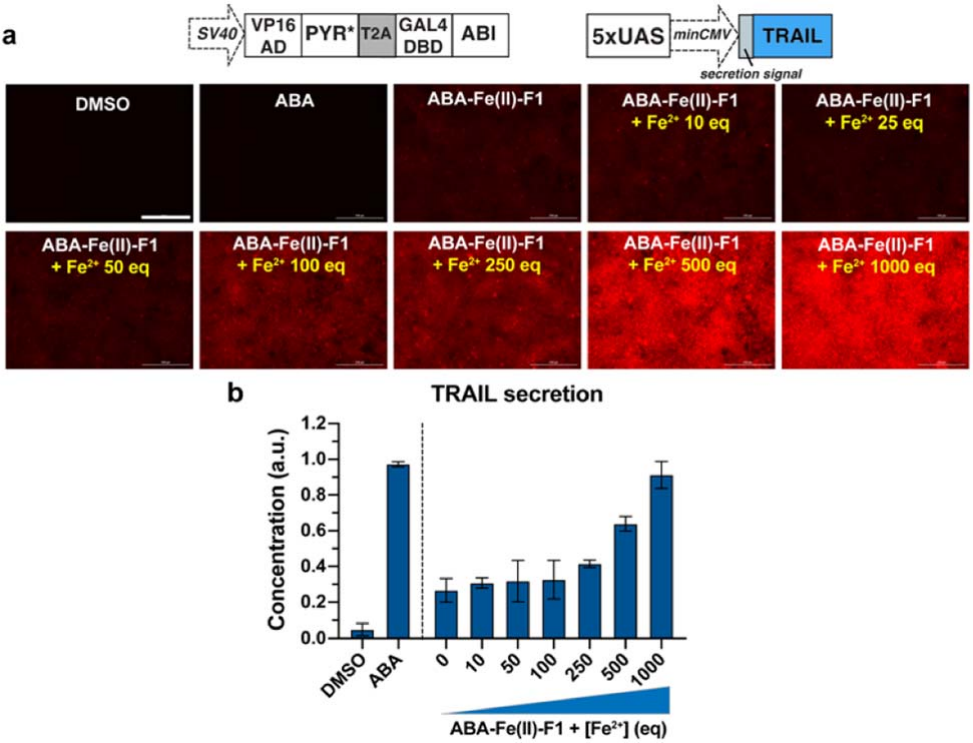
Fe^2+^-induced turn-on red fluorescence and secretion of sTRAIL in HEK-293T cells. (a) Cells were transfected with DNA constructs for inducible sTRAIL expression for 24 h and then treated with 10 μM ABA, 10 μM ABA-Fe(ii)-F1 or ABA-Fe(ii)-F1 with varying concentration of Fe^2+^, and the “turn-on” red fluorescence was readily detected at 30 min post Fe^2+^ treatment. Scale bars = 200 μm. (b) At 24 h post Fe^2+^ treatment, secreted TRAIL proteins in the culture media were quantified by the ELISA assay and normalized to ABA treatment. Data are mean ± SEM from 2 independent experiments performed in duplicate.

Next, to demonstrate the utilization of using ABA-Fe(ii)-F1 for theranostic purposes, we tested if Fe^2+^ can trigger the production of the tumor necrosis factor-related (TNF) apoptosis-inducing ligand (TRAIL), while reporting Fe^2+^ presence through turn-on fluorescence. TRAIL is a selective inducer of apoptosis in many transformed cells bearing the Death Receptor (DR) and has been a promising candidate for cancer therapies.^51–53^ An ABA-inducible expression of the secreted version of TRAIL (sTRAIL) can be controlled by the VP16AD-PYR*-T2A-GAL4DBD-ABI construct described above and an ABA inducible sTRAIL construct that we reported previously.^54^ HEK-293T cells were co-transfected with these plasmids for 24 h, and then treated with ABA-Fe(ii)-F1 (10 μM) in the presence or absence of Fe^2+^ at indicated concentrations following procedures as described above. At 30 min post Fe^2+^ treatment, red fluorescence signals were monitored using an automated fluorescence microscope. A dose-dependent increase in red fluorescence intensities with increasing Fe^2+^ concentration was observed (Fig. 9a). At 24 h after Fe^2+^ treatment, the secreted sTRAIL proteins in the culture media were quantified by the enzyme-linked immunosorbent assay (ELISA). We observed that the addition of Fe^2+^ induced the expression of secreted TRAIL and the increasing concentration of Fe^2+^ led to an increased level of sTRAIL expression (Fig. 9b). A low level of sTRAIL expression was detected when cells were incubated with ABA-Fe(ii)-F1 only, indicating that the cellular stability of the compound should be further optimized for applications requiring a prolonged incubation time. Taken together, these results established that the synthesized Fe^2+^-responsive ABA-Fe(ii)-F1 compound offers a promising direction in designing new dual functional theranostic molecular glues that can be combined with a highly customizable CIP platform to simultaneously report disease signals and induce desired therapeutic effects under various pathological conditions.

## Conclusion

In conclusion, we have demonstrated for the first time the integration of imaging and activation capacity within a molecular glue design to create a theranostic molecular glue. In this study, we have taken advantage of the rational design of a distinct carbamoyl oxime as a caging and a sensing unit by conjugating a NIR fluorophore DCM with ABA as a novel molecular glue. Notably, caging a ketone in ABA is a viable approach to control its activity. Furthermore, to enhance the sensitivity of the ABA-based molecular glues in inducing desired effects, we successfully engineered and enhanced the version of CIP with mutant PYR and ABI components that reduced the inducer-responding concentration from the μM to the nM range. We have shown that with the simultaneous activation of the fluorescence signal and molecular glue by a specific biological and disease relevant signal (*i.e.*, Fe^2+^), the presence of the signal of interest and the spatially controlled activation of desired biological effects and therapeutic interventions can be achieved. Although our theranostic molecular glue can get into cells, the increase of the molecular weight of theranostic molecular glues might compromise the cell permeability in new molecular glue structures. Therefore, their structural optimization is necessary. We envision that this novel theranostic molecular glue and integrated synthetic biology strategy provides an insight for designing similar theranostic molecular glues and holds great potential for precision therapies and biomedical research.

## Data availability

The datasets supporting this article have been uploaded as part of the ESI.†

## Author contributions

J. C., H. T. X. N., M. Y., F. Z. and H. X. designed, carried out, and analysed the experiments, and J. C. and H. N. wrote the manuscript draft. F.-S. L. and W. W. planned, designed, supervised, and directed the project, wrote and edited the manuscript, and acquired funding. All authors have given approval to the final version of the manuscript.

## Conflicts of interest

There are no conflicts to declare.

## Supplementary Material

SC-014-D2SC06995D-s001
