# Supplementary material for: A theranostic abscisic acid-based molecular glue
Source: Chem Sci. 2023 Feb 28;14(12):3377–84. doi: 10.1039/d2sc06995d (PMC10034047; doi:10.1039/d2sc06995d)
Supplement: SC-014-D2SC06995D-s001 [file SC-014-D2SC06995D-s001.pdf]

Electronic Supplementary Material (ESI) for Chemical Science.
This journal is © The Royal Society of Chemistry 2023

Supporting Information

A Theranostic Abscisic Acid-based Molecular Glue

Jing Chen,[a#] Huong Nguyen,[b#] Ming Yang, Fanxun Zeng,[a] Hang Xu,[a] Fu-Sen Liang,[b]* and Wei Wang[a]*

[a] *Departments of Pharmacology and Toxicology and Chemistry and Biochemistry, University of Arizona, Tucson, Arizona 85721, United States*

[b] *Department of Chemistry, Case Western Reserve University, 2080 Adelbert Road, Cleveland, Ohio 44106, United States*

Table of Contents

1. General information S2
2. Synthesis and characterization of probes S2
3. Characterization of theranostic probe ABA-Fe(II)-F1 in chemical system S3
4. NMR spectra S4
5. Cell lines and culture S5
6. Plasmid construction S5
7. Cellular localization of probe ABA-Fe(II)-F1 .... S6
8. Luciferase reporter assay S6
9. ABA-induced EGFP expression S6
10. Quantification of nuclear export in HEK-293T cells S7
11. Confocal fluorescence imaging S7
12. Fluorescence imaging experiments S7
13. ABA-induced secreted TRAIL and "turn-on" fluorescence S7
14. Flow cytometric analysis S8
15. Statistical analysis S8
16. Supplementary figures S9

1. General information

All reagents were purchased from commercial sources such as Sigma-Aldrich, Ambeed, and Fluka and used without further purification. The solvents were used by dry solvents system. All reactions were monitored by TLC or LC-MS from *Shimadzu*. Purification was conducted on preparative flash column chromatography and preparative reversed-phase high performance liquid chromatography (RP-HPLC) with solvent systems specified. Nuclear magnetic resonance (NMR) spectra were recorded on automated Bruker AVIII-400 instruments. High-resolution mass spectra (HRMS) were recorded on a Bruker microTOF II instrument in positive ion mode using an Agilent G1969 API-TOF with an electrospray ionization (ESI) source. Ultraperformance liquid chromatography (UPLC) spectra for compounds were acquired using a *Shimadzu* LabSolutions system.

2. Synthesis and characterization of probes

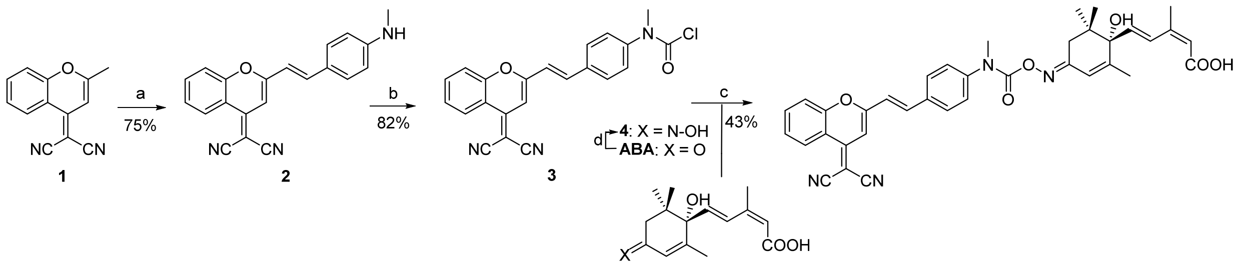

[a]Reagents and conditions: (a) 4-(methylamino)benzaldehyde, AcOH, Piperidine, PhMe, reflux, 5h; (b) Triphosgene, DCM, r.t., 12h; (c) **5b**, Pyridine. (d) hydroxylamine, MS, Pyridine, reflux, N_2, 12h.

(*E*)-2-(2-(4-(Methylamino)styryl)-4H-chromen-4-ylidene)malononitrile (2). Compound **1** (1.04 g, 5 mmol), and 4-(methylamino)benzaldehyde (0.67 g, 5 mmol) were dissolved in toluene (60 mL) with piperidine (2.5 mL) and acetic acid (2.5 mL) at room temperature. Then the mixture was refluxed for 3 h using a Dean-Stark apparatus to remove water. Then the mixture was cooled to room temperature and formed a red precipitate. After filtration and washing with CH_2Cl_2, compound **3** (1.21 g, 75%) was obtained. ^{1}H NMR (500 MHz, DMSO) δ 8.72 (dd, *J* = 8.3, 1.4 Hz, 1H), 7.91 - 7.86 (m, 1H), 7.77 (dd, *J* = 8.5, 1.3 Hz, 1H), 7.66 (d, *J* = 15.7 Hz, 1H), 7.60 - 7.53 (m, 3H), 7.10 (d, *J* = 15.7 Hz, 1H), 6.86 (s, 1H), 6.62 - 6.57 (m, 3H), 2.76 (d, *J* = 4.9 Hz, 3H). ^{13}C NMR (126 MHz, DMSO) δ 160.3, 153.0, 152.9, 152.6, 141.1, 135.5, 131.1, 126.4, 125.0, 122.7, 119.4, 118.3, 117.7, 117.0, 112.9, 112.2, 105.2, 57.7, 29.7.

(*E*)-(4-(2-(4-(Dicyanomethylene)-4H-chromen-2-yl)vinyl)phenyl)(methyl)carbamic chloride (3). Compound **2** (1.0 g, 3 mmol) and pyridine (474.6 μL, 6 mmol) were dissolved in CH_2Cl_2 (10 mL) and cooling to 0 °C. Triphosgene (445 mg, 1.5 mmol) was added, and the reaction was stirring at 0-5 °C for 2 h. Warm the reaction mixture to room temperature and keep stirring for further 2 h. Monitor the consumption of the starting material by TLC. Dilute the reaction with CH_2Cl_2 (20 mL) and wash with 1 M HCl, adequate brine. Dry the organic layer over sodium sulfate and concentrate the organic layer under vacuum to give yellow solid power compound 4 (952 mg, 82%).

ABA-Fe(II)-F1. ABA (100 mg, 0.38 mmol), hydroxylamine (13.2 mg, 0.4 mmol) and molecular sieve (100 mg) were added to pyridine solvent (5 ml) under the nitrogen atmosphere. The reaction was heated to reflux overnight. Cool down the reaction to room temperature and filtered out the molecular sieve. To the mixture of ABA oxime **4**, compound **3** (193 mg, 0.5 mmol) was added and allow the reaction to stir at room temperature for 2 h. The product was purified by prep-HPLC with methanol and water as the solvent and lyophilized to give yellow power ABA-Fe(II)-F1 (102 mg, 43%). ^{1}H NMR (500 MHz, CD_3OD) δ 8.80-8.77 (m, 1H), 7.83-7.79 (m, 1H), 7.74-7.64 (m, 5H), 7.49-7.45 (m, 1H), 7.43-7.39 (m, 2H), 7.10-7.07 (7.08-7.05) (d, J = 15.9 Hz, 1H), 6.87 (6.86) (s, 1H), 6.23 (6.12) (s, 1H), 6.16-6.13 (6.11-6.08) (d, J = 16.0 Hz, 1H), 5.72 (5.70) (s, 1H), 3.39 (3.38) (s, 3H), 2.01 (1.99) (s, 3H), 1.85 (1.79) (s, 3H), 1.00 (0.97) (s, 3H), 0.94 (0.93) (s, 3H). ^{13}C NMR (126 MHz,, CD_3OD) 169.40, 162.16, 159.33, 157.59, 155.58 (155.54), 154.27 (153.78), 151.26 (151.23), 145.62 (145.55), 139.11, 138.68 (138.58), 136.31, 134.67, 129.96 (129.91), 129.20 (128.88), 127.45 (127.37), 127.05, 126.36, 120.49 (120.47), 120.31, 120.06, 119.30 (119.25), 118.72, 117.91, 116.60, 115.44, 107.90, 80.65, 62.88, 41.13, 40.19 (39.89), 37.91 (37.44), 24.65 (24.53), 23.80 (23.03), 21.32 (21.27), 20.03 (19.31). LRMS calcd. For $C_{37}H_{34}N_4O_6$ $[M + H]^+$ 631.2557 found 631.2551.

3. Characterization of theranostic probe ABA-Fe(II)-F1 in chemical system

All aqueous solutions were prepared using Milli-Q water, and all spectroscopic experiments were carried out in 50 mM HEPES, pH 7.4, unless otherwise noted. All spectroscopic experiments were carried out using freshly prepared aliquots, unless otherwise noted. Absorption spectra were acquired on a Varian Cary 50 spectrophotometer, and fluorescence spectra were acquired using fluorescence spectrometer a 1 cm × 1 cm quartz cuvettes (1.4 mL volume, Starna, capped) were used for obtaining absorption and fluorescence spectra. For all fluorescence response to iron(II) studies, aqueous solutions of $Fe(NH_4)_2(SO_4)_2(H_2O)_6$ (FAS) (Sigma) were used. For metal selectivity studies, aqueous metal solutions of NaCl, KCl, $CaCl_2{\cdot}2H_2O$, $MgCl_2{\cdot}$ $4H_2O$, $FeCl_3$, $ZnCl_2$, $NiCl_2{\cdot}$ $6H_2O$, $MnCl_2{\cdot}4H_2O$, $CuCl_2{\cdot}2H_2O$, $[Cu(CH_3CN)_4]PF_6$, and $CoCl_2{\cdot}6H_2O$ were used. GSH, Lysine and Cysteine were used for selectivity studies.

Fluorescence responses studies of ABA-Fe(II)-F1 to Fe^{2+}. A 10 μM solution of ABA-Fe(II)-F1 (990 μL) was prepared by diluting a 10 mM DMSO stock solution of ABA-Fe(II)-F1 into 50% HEPES/DMSO (10 mM, pH 7.4) in a 1 cm × 1 cm capped quartz cuvette. The probe solution was incubated at 37 °C for 5 min, then 10 μL of 10 mM stock solution of ferrous ammonium sulfate (FAS) (freshly prepared by diluting FAS into Milli-Q water) was added to yield a final concentration of 100 μM. The mixture was then vortexed in the capped cuvette, then the t = 0 spectrum was acquired. Emission spectra (λ_{ex} = 450 nm, λ_{em} = 550-900 nm) were collected at t = 0, 1, 2, 3, 4, 6, 8, 10, 15, 30 min. Temperature was maintained at 37 °C throughout the experiment by incubating cuvettes in a heated water bath.

Sensitivity studies of ABA-Fe(II)-F1. 990 μL of 10 μM solution of ABA-Fe(II)-F1 was prepared by diluting a 10 mM DMSO stock solution of ABA-Fe(II)-F1 into 50% HEPES/DMSO (10 mM, pH 7.4) in a 1 cm × 1 cm capped quartz cuvette. The probe solution was incubated at 37 °C for 5 min. Then 10 L of a 0.1, 0.2, 0.4, 0.6, 0.8, 1, 2, 4, 6, 8, 10 mM FAS (freshly prepared by diluting

FAS into Milli-Q water) was added to yield a final concentration of 1, 2, 4, 6, 8, 10, 20, 40, 60, 80, 100 µM. The mixture was then vortexed in the capped cuvette, then the t = 0 spectrum was acquired. Emission spectra (λ_{ex} = 450 nm, λ_{em} = 550-900 nm) were collected. Temperature was maintained at 37 ºC throughout the experiment by incubating cuvettes in a heated water bath.

Selectivity studies of ABA-Fe(II)-F1 toward metal ions, GSH, lysine and cysteine. 10 mL of 10 µM solution of ABA-Fe(II)-F1 was prepared by diluting a 10 mM DMSO stock solution of ABA-Fe(II)-F1 into 50% HEPES/DMSO (10 mM, pH 7.4) in a 1 cm × 1 cm capped quartz cuvette. 500 µL of this solution were added to ten 1 cm × 1 cm capped quartz cuvettes, then the cuvettes were placed in a 37 °C water bath for 5 min. After 5 min, 500 µL of a solution of the metal of interest was added to the cuvette to bring the concentration of transition metals to 100 µM and the concentration of NaCl, KCl, $CaCl_2 \cdot 2H_2O$, $MgCl_2 \cdot 4H_2O$ to 1 mM, final GSH, Lysine and Cysteine concentration of 5 mM. 500 µL of buffer was added to one cuvette, and this sample served as the blank throughout the experiment.

4. NMR spectra

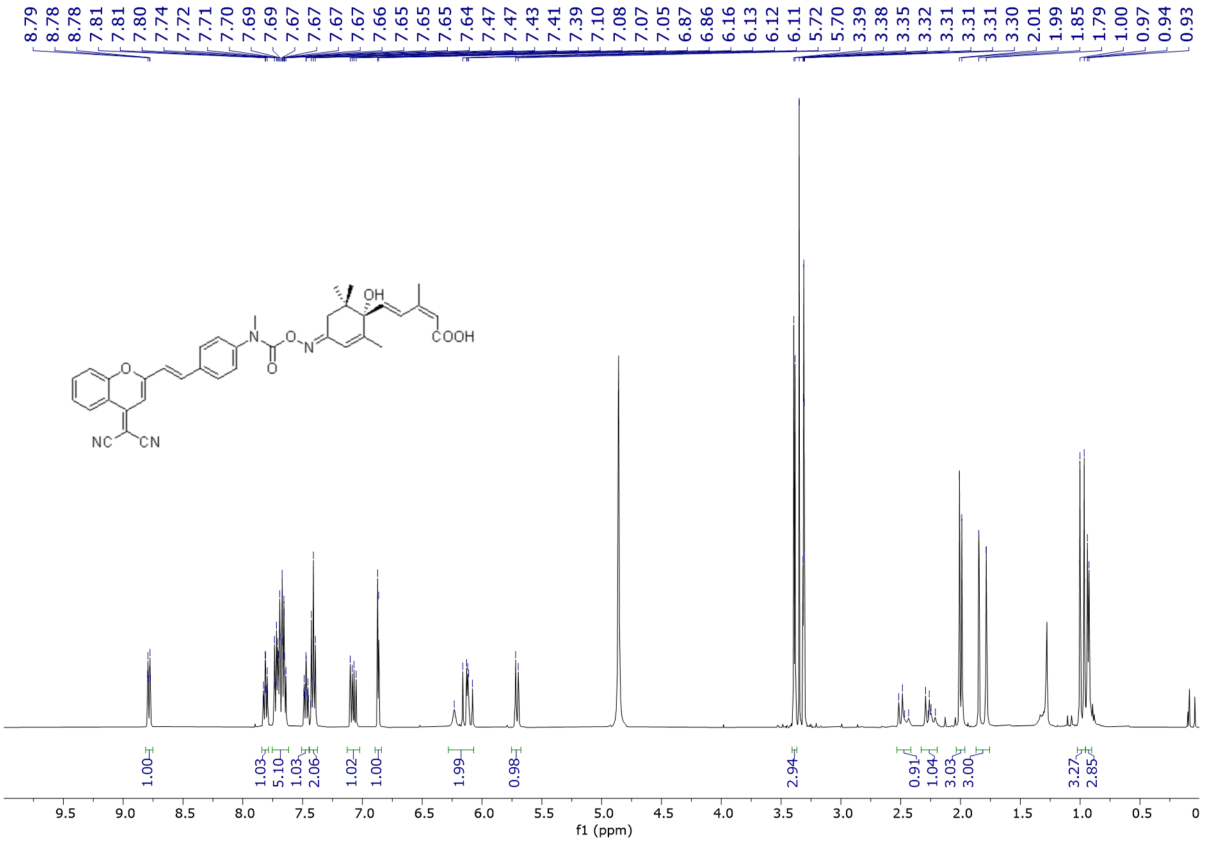

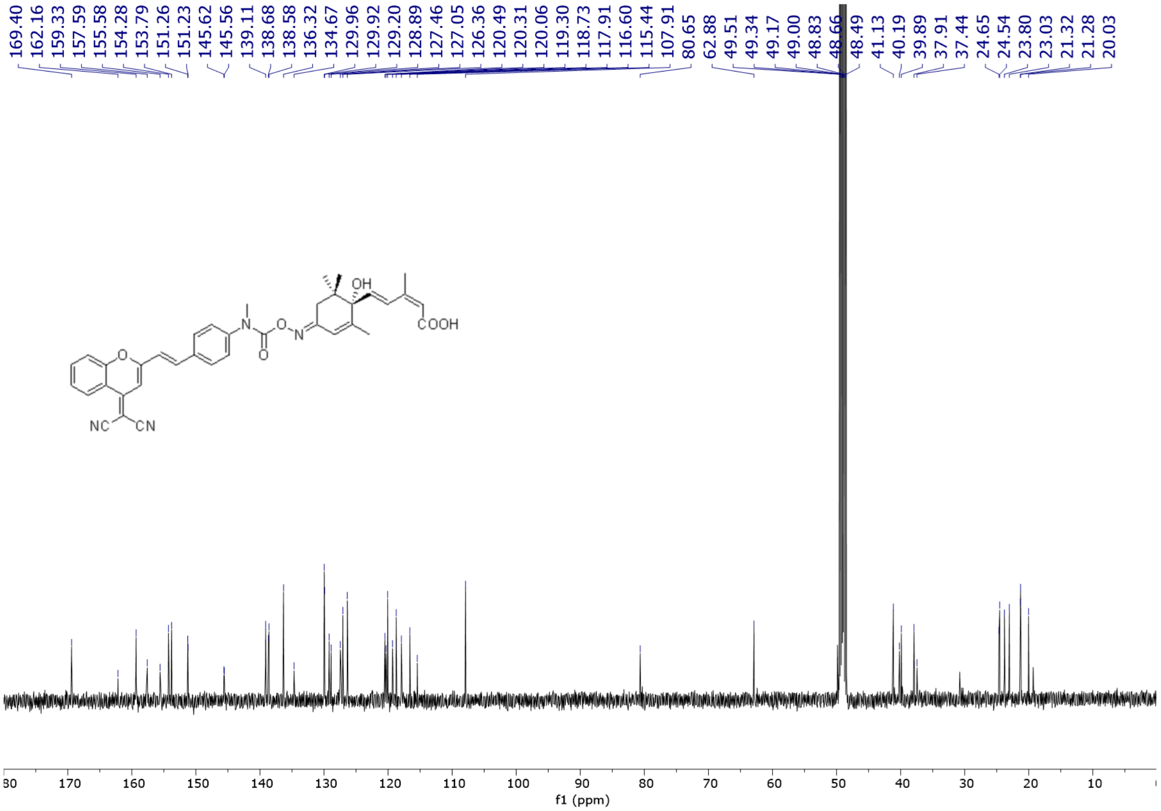

5. Cell lines and culture. Human embryonic kidney cells (HEK-293T) were obtained from Dr. Alex Huang's lab (CWRU School of Medicine). Human cervical cancer (HeLa) cells, Chinese Ovary Hamster (CHO) cells, human breast cancer cells (MDA-MB-231) were maintained in our laboratory. Cells were grown in Dulbecco's modified Eagle's medium (DMEM) (Gibco) supplemented with 10%(v/v) heat-inactivated fetal bovine serum (FBS, Omega Scientific), 5%(v/v) GlutaMAX (Life Technologies) and 100 IU/ml penicillin/streptomycin (Life Technologies) in a humidified 37ºC incubator with 5% CO_2 atmosphere. Cells were tested for mycoplasma and confirmed mycoplasma-free using MycoAlert™ mycoplasma detection kit (Lonza).
For all cell culture experiment, stock solution of ABA and theranostic compound were prepared in DMSO. A 500 mM of stock Fe^{2+} solution was prepared by mixing ferrous chloride (Sigma) and sodium citrate (Thermofisher) at 1:1 molar ratio. Fresh Fe^{2+} solution was made before every experiment.

6. Plasmid construction. Construction of the ABA-split transcriptional activator construct sv40-vp16-PYL-ires-Gal4DBD-ABI_{D134A}(sv-VPiGA) has been described previously.[2] Three mutant PYR1 versions including E141L, F61L/A160C and F61L/E141L/A160V mutations were synthesized as gBlock gene fragments and cloned into the sv-VPiGA to replace PYL1, using infusion cloning (Takara). The optimized version sv-VP*2GA construct was modified from the current sv-VPiGA by replacing PYL with $PYR_{F61L/A160C}$ and IRES with a self-cleavable linker T2A peptide derived from foot-and-mouth disease virus.

The nuclear export plasmid cassette including NES-ABI and EGFP-PYL was described previously.[3] The modified expression vector EGFP-PYR* was made by replacing PYL from the parental EGFP-PYL with $PYR_{F61L/A160C}$ using in-fusion cloning (Takara). PYR* was PCR amplified using the sv-VP*2GA construct as a template with respective forward and reversed primers (5'-TGGACGAGCTGTACAAGGGCGCGCCACCATCAGAGCTTACCCCCGA-3' and 5'-TGATTATGATCTAGAGTCGCGGCCGCTCAAGCGTAATCTGGAACATCGT-3') and inserted into the linearized Actin-EGFP vector with AscI and NotI restriction sites.

The inducible TRAIL plasmid was constructed by replacing EGFP gene with the nucleotide sequence encoding IL2Rα secretion signal and TRAIL (aa 114-281) under control of a minimal CMV promoter and 5xUAS response element.

All plasmid constructs were amplified using DH5α chemically competent E.coli strain and purified using Endo-free Qiagen Miniprep kit. DNA sequences of the final constructs were confirmed by Sanger sequencing (Genewiz). Plasmid concentration and purity (A 260/280 = 1.88-1.90) were measured by a Nanodrop spectrophotometer (Thermofisher).

7. Cellular localization of probe ABA-Fe(II)-F1. The subcellular localization of probe was tested on three different cell lines including HEK-293T, HeLa, and MDA-MB-231. The cells were seeded into a 96-well black wall clear bottom plate 1 day prior to drug treatment. On the day of imaging, cells were treated with 10 μM ABA-Fe(II)-F1 in the presence of mixture of 5 mM $FeCl_2$ and 5 mM sodium citrate for 30 min. Media were removed and cells were stained with NucBlue™ Live ReadyProbes™ Reagent (Hoechst 33342, Invitrogen) and either 6 μM ER-Tracker® Green (Cell Signaling Technology) or 500 nM LysoTracker® Green (Cell Signaling Technology) for 20 min. Stained cells were then washed twice with DPBS, submerged in DPBS and imaged immediately by Leica HyVolution SP8 confocal microscope using 20x-water immersion objective.

8. Luciferase reporter assay. 10^4 CHO cells were seeded in triplicate in a 96-well plate and allowed to adhere for 24 h at 37°C in a CO_2 incubator. Cells were transfected with ABA-responsive split transcriptional activator with PYL1 being replaced with PYR1 mutants including E141L, F61L/A160C and F61L/E141L/A160V mutations, and 5xUAS response elements controlling the expression of firefly luciferase. 24 h post-transfection, cells were incubated with varying concentration of ABA or DMSO alone (as control). Luciferase activity was detected using Luciferase Assay System (Promega #E1500), for luminometer (GLOMAX-Multi Detection System) according to manufacturer's protocol. The triplicate data obtained for each mutant condition were averaged and normalized to DMSO treatment.

9. ABA-induced EGFP expression. 10^4 HEK-293T cells were seeded in duplicate in a 96-well plate and allowed to adhere for 24 h at 37°C in a humidified CO_2 incubator. Cells were transfected, using PEI, with the optimized ABA-responsive split transcriptional activator and an inducible EGFP expression. 24 hours after transfection, the cells were incubated with either DMSO, 10 μM ABA or 10 μM ABA-Fe(II)-F1. One hour later, the cell culture media were changed with fresh DMEM, and varying concentrations of Fe^{2+} were added to the cells treated with ABA-Fe(II)-F1 to uncage the compound and generate free ABA. Expression of EGFP was monitored intermittently at 6, 12 and 24 h.

10. Quantification of nuclear export in HEK-293T cells. Two days before imaging, cells were seeded on 17-mm glass coverslips in a 24-well plate at 50,000 cells/well in a total volume of 500 µL DMEM media and incubated overnight. Transfection was performed using NES-ABI and EGFP-PYR* plasmids at a ratio of 2:1 in PEI transfection reagent (Polyscience). Fresh media was replaced at 6 hours post transfection. 16 h after transfection, cells were treated with either 10 µM ABA, 10 µM ABA-Fe(II)-F1, or 10 µM ABA-Fe(II)-F1 with added 500 eq. of Fe^{2+} (prepared in Sodium citrate stock solution at 500 mM). At indicated time points, media were removed, and cells were stained with NucBlue™ Live ReadyProbes™ Reagent (Hoechst 33342, Invitrogen) for 30 min at 37°C in the dark. Cells were then washed twice with PBS and fixed in 4% formaldehyde fixation solution (Thermofisher) for 10 min at room temperature. Following fixation, cells were washed twice with PBS (5 min each) and mounted on a glass microscopic slide with Vectashield anti-fade mounting media. Samples were stored at 4°C until ready to use.

To quantify the nuclear translocation, mean green fluorescence intensity of the nuclear and cytoplasmic regions were calculated using ImageJ software. Background-subtracted images were quantified by defining ROI of the nuclear and cytoplasmic regions of each cell for a population of 20-30 cells. The reported nuclear translocation is the ratio between the mean fluorescence intensities of the nuclear (Fn) and cytoplasmic (Fc) regions.

11. Confocal fluorescence imaging. Confocal fluorescence imaging was performed with Leica HyVolution SP8 confocal microscope equipped with 20x- water immersion, 40x- and 63x-oil immersion objective lens. Relevant quantifications were performed by the ImageJ software. Red fluorescence (Probe) was detected using Hybrid photodetector at 514 nm excitation and emission was collected at 685-720 nm excitation window. EGFP-expressed cells were excited with a 488 nm PMT laser and emission was collected between 500-550 nm. Excitation of Hoechst 33342 at 405 nm was carried out with diode laser and emission was collected between 415-485 nm. ER-Tracker and LysoTracker dye were excited at 496 nm with argon laser, and the emission was collected between 510-560 nm. The bright field images of the cells were taken with a transmitted light photomultiplier under a 514-nm laser.

12. Fluorescence imaging experiments. Cells were imaged using Lionheart™ FX Automated Microscope (BioTek Instruments, Winooski, VT) configured with DAPI, GFP, and Cy5 filter cubes. Hoechst 33342 was detected with a 337/50 excitation filter and a 447/60 emission filter. The GFP light cube uses a 469/35 excitation filter and a 525/39 emission filter. Red fluorophore was detected using a Cy5 filter cube with a 628/50 excitation filter and a 685/50 emission filter.

13. ABA-induced secreted TRAIL and "turn-on" fluorescence. HEK-293T cells were dual-transfected with sv-VP*2GA and the inducible plasmid encoding secreted TRAIL (sTRAIL) under control of minimal CMV promoter and 5xUAS response element. At 24 h post-transfection, cells were treated with either DMSO or 10 µM ABA-Fe(II)-F1. 1 hour later, media were refreshed and varying concentrations of Fe^{2+} were then added to corresponding wells, 10 µM ABA was also added to the control well. At 15-minute post-treatment, cells were imaged with Lionheart™ FX Automated Microscope using 628/50 excitation filter and 685/50 emission filter for the generation of probe. Cells were then transferred back into the incubator and continue to grow overnight. At

24 h post-iron treatment, cell culture supernatant was collected and quantified for the secretion of TRAIL using human TRAIL PicoKine^{TM} ELISA kit (Boster Bio, Pleasanton, CA).

14. Flow cytometric analysis. Cells for each experimental condition were harvested by trypsinization, washed with DPPS, resuspended in DPBS, then subjected to analysis by flow cytometry using BD Accuri™ C6 Plus Flow Cytometer (BD Biosciences) equipped with a Blue (488 nm) and a Red (640 nm) lasers. Green fluorescent signals were detected on FITC channel and red fluorescent signals were detected on PerCP channel. Data were analyzed with FlowJo software (v10, TreeStar).

15. Statistical analysis.
Statistical significance was calculated in GraphPad Prism using two tailed unpaired Student's t-test for two independent groups, and ANOVA for multiple groups comparison. All data are presented as mean±S.E.M.

16. Supplementary figures.

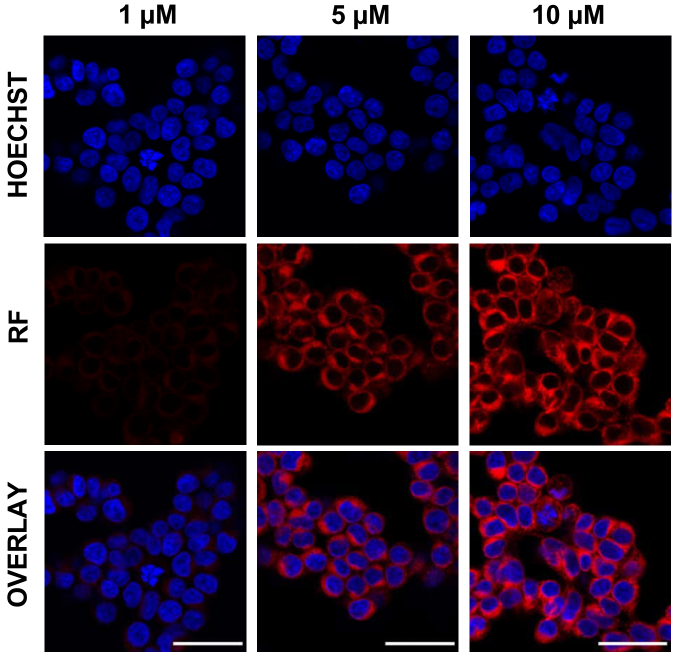

Figure S1. HEK-293T cells treated with different concentrations of ABA-Fe(II)-F1 in the presence of 5 mM Fe^{2+} for 30 min. The turn-on fluorescence (the RF panel) was shown in red (Ex/Em = 514 nm/(685-720 nm)) and cell nuclei were stained with Hoechst shown in blue (Ex/Em= 405 nm/(415-485 nm)). Scale bars = 40 μM. Shown were representative images from 3 independent experiments.

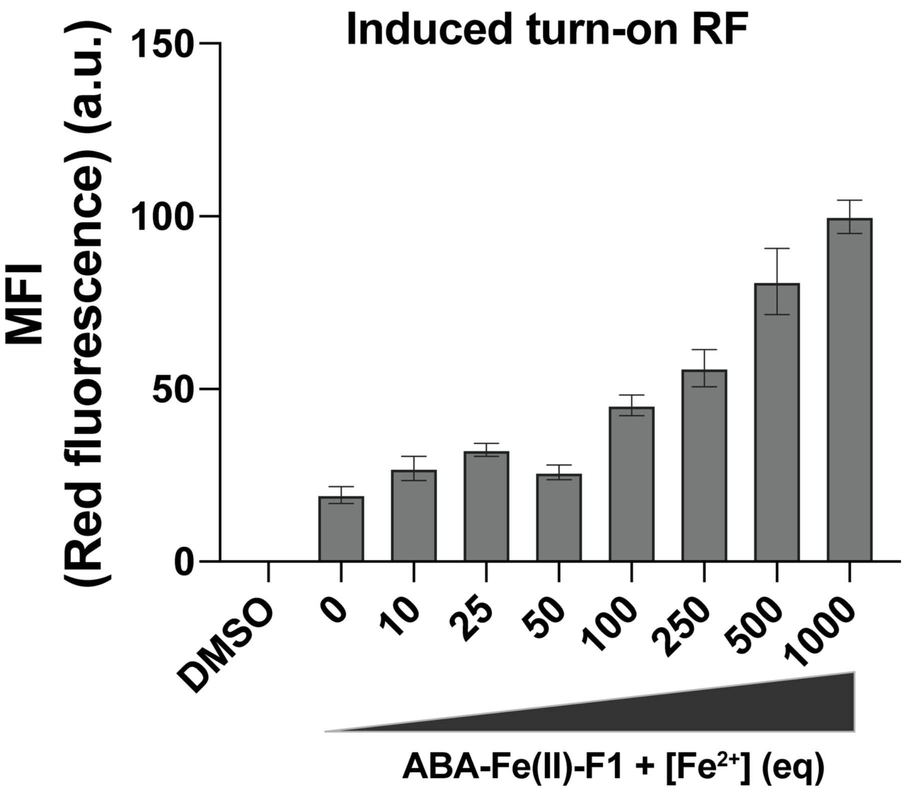

Figure S2: Quantification of dose-dependent turn-on red fluorescence from confocal images. The mean red fluorescence intensities in cells from the confocal images were quantified by ImageJ software. Data shown represent the mean ± S.E.M (n=4-5).

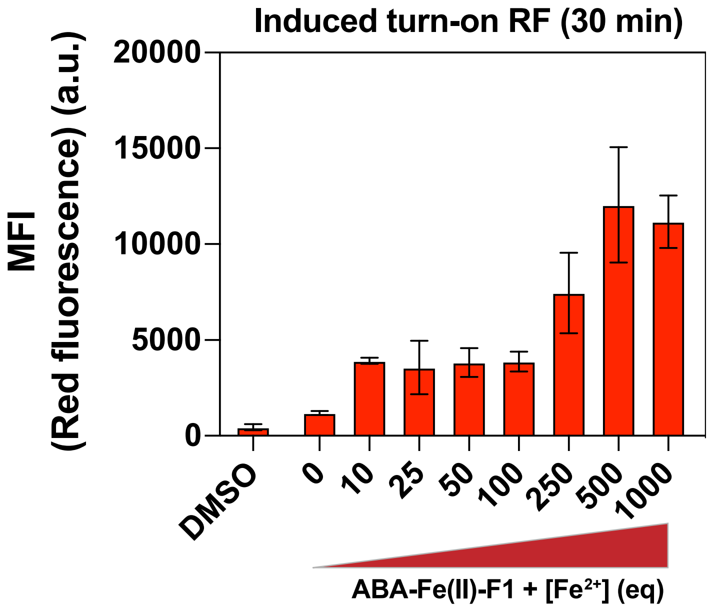

Figure S3. Flow cytometric quantification of dose-dependent turn-on red fluorescence. HEK-293T cells were incubated with 10 μM ABA, ABA-Fe(II)-F1 or ABA-Fe(II)-F1 plus varying concentrations of Fe^{2+} for 30 minutes. 10^4 cells were then collected and analyzed by flow cytometry. Error bars represent mean± S.E.M from independent biological experiments (n=3).

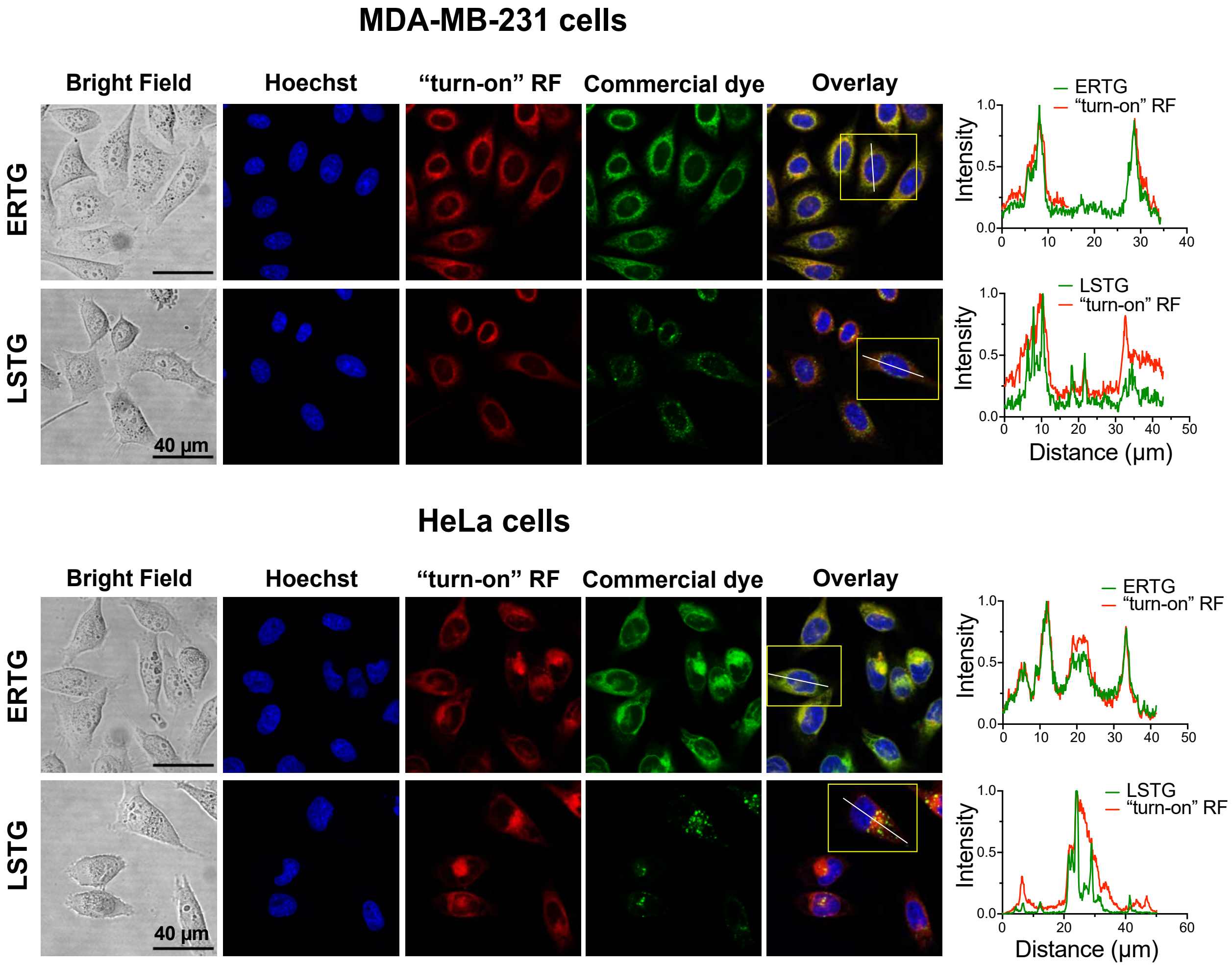

Figure S4. **Co-localization of the turn-on fluorescence probe DCM released from ABA-Fe(II)-F1 with ER or lysosome dyes in MDA-MB-231 and HeLa cells.** Cells were treated with ABA-Fe(II)-F1 (10 μM) and Fe^{2+} (5 mM) for 30 min and co-stained with Hoechst and ERTG or LSTG. Overlay images shown merging of Hoechst, generated red fluorescence from DCM (the RF panel) and commercial ER and lysosome dye channels. A representative intensity profile of the generated red fluorescence and each dye from ROI were shown on the right. Imaging channels: Hoechst: E_x=405 nm/E_m=[415-485 nm]; Red fluorescence: E_x= 514 nm/E_m=[685-720] nm; ERTG and LSTG: E_x=496 nm/E_m=[510-560 nm]. A scale bar of 40 μm is shown for all images. Shown were representative images from 3 independent experiments.

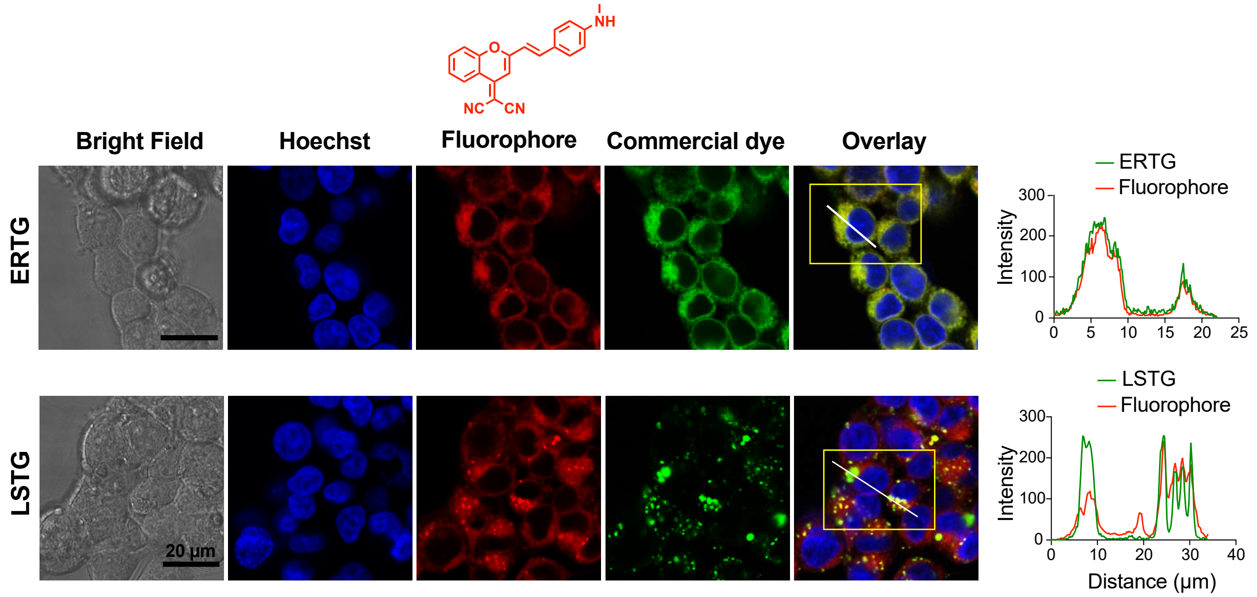

Figure S5. Confocal images showing co-localization of the DCM fluorophore with commercial ER and lysosome dyes in HEK-293T cells. Cells were co-stained with 10 µM free DCM fluorophore, Hoechst and ERTG or LSTG for 30 min, then washed and imaged. Overlay images shown merging of Hoechst, fluorophore (DCM) and commercial dye channels. A representative intensity profile of the fluorophore and each dye from ROI were shown on the right. Imaging channels: Hoechst: E_x=405 nm/E_m=[415-485 nm]; Fluorophore: E_x= 514 nm/E_m=[685-720] nm; ERTG and LSTG: E_x=496 nm/E_m=[510-560 nm]. A scale bar of 20 µm is shown for all images. Shown were representative images from 2 independent experiments.

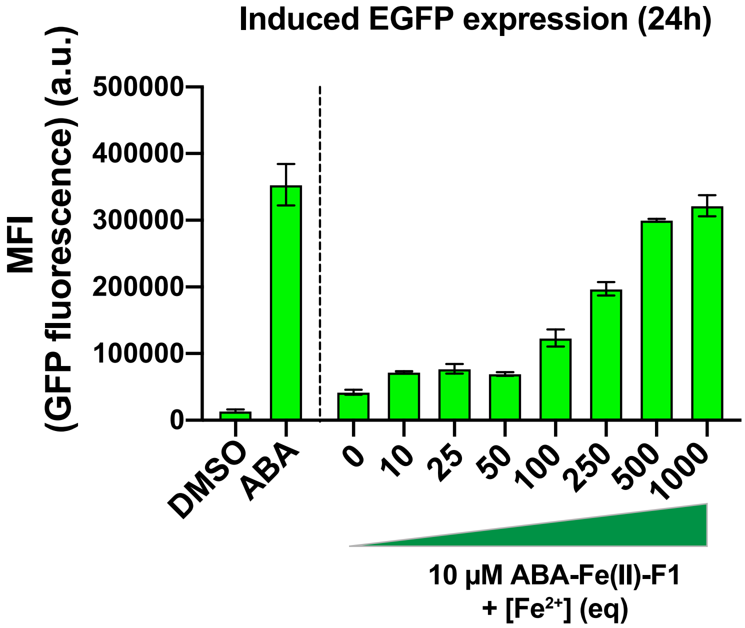

Figure S6. The quantification of induced-EGFP expression using flow cytometry. 10 µM of ABA, ABA-Fe(II)-F1 or ABA-Fe(II)-F1 plus varying concentration of Fe^{2+} were added to transfected HEK-293T cells as described in **Figure 7**. At 24 h, 10^4 cells were analyzed by flow cytometry. Error bars are mean ± S.E.M (n=2)

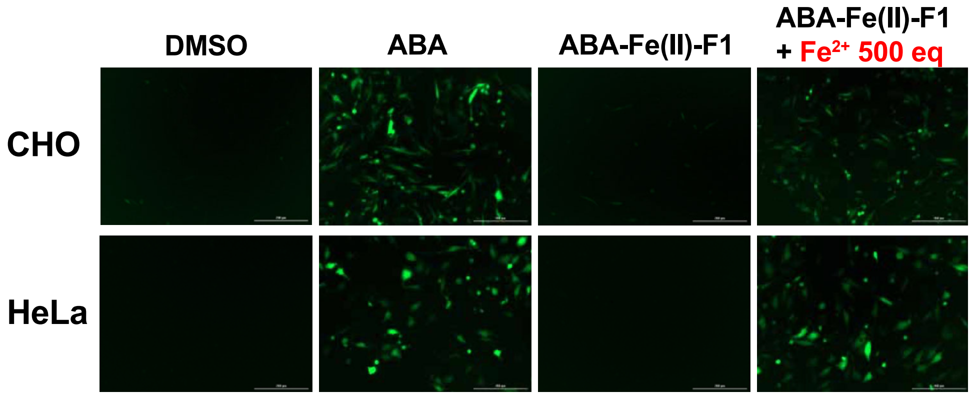

Figure S7. Fe^{2+}-induced EGFP expression in CHO and HeLa cells. Cells were transfected with plasmids of inducible EGFP-expression and the ABA-responsive split transcriptional activator containing PYR* for 24 h, and then treated with 1 μM ABA, 1 μM ABA-Fe(II)-F1, or 1 μM ABA-Fe(II)-F1 with added Fe^{2+} (500 μM). Images were taken using an automated fluorescence microscope. Shown were representative images from 3 independent experiments.
